# Incidence of Stress Cardiomyopathy During the Coronavirus Disease 2019 Pandemic

**DOI:** 10.1001/jamanetworkopen.2020.14780

**Published:** 2020-07-09

**Authors:** Ahmad Jabri, Ankur Kalra, Ashish Kumar, Anas Alameh, Shubham Adroja, Hanad Bashir, Amy S. Nowacki, Rohan Shah, Shameer Khubber, Anmar Kanaa’N, David P. Hedrick, Khaled M. Sleik, Neil Mehta, Mina K. Chung, Umesh N. Khot, Samir R. Kapadia, Rishi Puri, Grant W. Reed

**Affiliations:** 1 Department of Internal Medicine, Cleveland Clinic Akron General, Akron, Ohio; 2 Department of Cardiovascular Medicine, Heart, Vascular and Thoracic Institute, Cleveland Clinic, Cleveland, Ohio; 3 Heart, Vascular and Thoracic Department, Cleveland Clinic Akron General, Akron, Ohio; 4 Department of Critical Care Medicine, St John’s Medical College Hospital, Bengaluru, India; 5 Department of Quantitative Health Sciences, Lerner Research Institute, Cleveland Clinic, Cleveland, Ohio; 6 Department of Hospital Medicine, Cleveland Clinic, Cleveland, Ohio; 7 Lerner College of Medicine, Cleveland Clinic, Department of Medicine, Case Western Reserve University, Cleveland, Ohio

## Abstract

**Importance:**

The coronavirus disease 2019 (COVID-19) pandemic has resulted in severe psychological, social, and economic stress in people’s lives. It is not known whether the stress of the pandemic is associated with an increase in the incidence of stress cardiomyopathy.

**Objective:**

To determine the incidence and outcomes of stress cardiomyopathy during the COVID-19 pandemic compared with before the pandemic.

**Design, Setting, and Participants:**

This retrospective cohort study at cardiac catheterization laboratories with primary percutaneous coronary intervention capability at 2 hospitals in the Cleveland Clinic health system in Northeast Ohio examined the incidence of stress cardiomyopathy (also known as *Takotsubo syndrome*) in patients presenting with acute coronary syndrome who underwent coronary arteriography. Patients presenting during the COVID-19 pandemic, between March 1 and April 30, 2020, were compared with 4 control groups of patients with acute coronary syndrome presenting prior to the pandemic across 4 distinct timelines: March to April 2018, January to February 2019, March to April 2019, and January to February 2020. Data were analyzed in May 2020.

**Exposures:**

Patients were divided into 5 groups based on the date of their clinical presentation in relation to the COVID-19 pandemic.

**Main Outcomes and Measures:**

Incidence of stress cardiomyopathy.

**Results:**

Among 1914 patient presenting with acute coronary syndrome, 1656 patients (median [interquartile range] age, 67 [59-74]; 1094 [66.1%] men) presented during the pre–COVID-19 period (390 patients in March-April 2018, 309 patients in January-February 2019, 679 patients in March-April 2019, and 278 patients in January-February 2020), and 258 patients (median [interquartile range] age, 67 [57-75]; 175 [67.8%] men) presented during the COVID-19 pandemic period (ie, March-April 2020). There was a significant increase in the incidence of stress cardiomyopathy during the COVID-19 period, with a total of 20 patients with stress cardiomyopathy (incidence proportion, 7.8%), compared with prepandemic timelines, which ranged from 5 to 12 patients with stress cardiomyopathy (incidence proportion range, 1.5%-1.8%). The rate ratio comparing the COVID-19 pandemic period to the combined prepandemic period was 4.58 (95% CI, 4.11-5.11; *P* < .001). All patients during the COVID-19 pandemic had negative reverse transcription–polymerase chain reaction test results for COVID-19. Patients with stress cardiomyopathy during the COVID-19 pandemic had a longer median (interquartile range) hospital length of stay compared with those hospitalized in the prepandemic period (COVID-19 period: 8 [6-9] days; March-April 2018: 4 [3-4] days; January-February 2019: 5 [3-6] days; March-April 2019: 4 [4-8] days; January-February: 5 [4-5] days; *P* = .006). There were no significant differences between the COVID-19 period and the overall pre–COVID-19 period in mortality (1 patient [5.0%] vs 1 patient [3.6%], respectively; *P* = .81) or 30-day rehospitalization (4 patients [22.2%] vs 6 patients [21.4%], respectively; *P* = .90).

**Conclusions and Relevance:**

This study found that there was a significant increase in the incidence of stress cardiomyopathy during the COVID-19 pandemic when compared with prepandemic periods.

## Key Points

**Question:**

Is psychological, social, and economic stress associated with coronavirus disease 2019 (COVID-19) associated with the incidence of stress cardiomyopathy?

**Findings:**

This cohort study included 1914 patients with acute coronary syndrome to compare patients presenting during the COVID-19 pandemic with patients presenting across 4 timelines prior to the pandemic and found a significantly increased incidence of 7.8% of stress cardiomyopathy during the COVID-19 pandemic, compared with prepandemic incidences that ranged from 1.5% to 1.8%.

**Meaning:**

These findings suggest that psychological, social, and economic stress related to the COVID-19 pandemic was associated with an increased incidence of stress cardiomyopathy.

## Teaser

This cohort study compares the incidence of stress cardiomyopathy among patients presenting with acute coronary syndrome at 2 Ohio hospitals during the coronavirus disease 2019 pandemic vs prepandemic time points.

## Introduction

The World Health Organization has declared coronavirus disease 2019 (COVID-19), caused by the severe acute respiratory syndrome coronavirus 2, a global pandemic. As of June 2020, nearly 8.5 million infections have been reported worldwide, resulting in approximately 450 000 deaths in more than 200 countries and territories. The effect of COVID-19 has extended beyond health care, having significant social, economic, and cultural ramifications. The global effects of the virus have been linked with increasing stress and anxiety worldwide.^1^ Recently, clinicians have reported a rise in stress cardiomyopathy (also known as *Takotsubo syndrome*) worldwide during the COVID-19 pandemic.^2,3^ This observation warrants further investigation to unravel a plausible pathogenic mechanism associated with COVID-19 causing Takotsubo syndrome–like cardiomyopathy vs a true increase in its incidence due to the associated psychological, social, and economic stress with imposed quarantine, lack of social interaction, strict physical distancing rules, and its economic consequences in people’s lives. Our study investigated the incidence of stress (ie, Takotsubo) cardiomyopathy during the COVID-19 pandemic in comparison with its incidence in historical cohorts, its association with the viral infection, and related outcomes.

## Methods

This cohort study was approved by the institutional review board at Cleveland Clinic. A waiver of informed consent was granted because of minimal risk exposure to patients per Cleveland Clinic policy. This study followed the Strengthening the Reporting of Observational Studies in Epidemiology (STROBE) reporting guideline.

### Study Population

We retrospectively analyzed the electronic medical records of all patients presenting with acute coronary syndrome (ACS) (ie, ST-segment elevation myocardial infarction, non–ST-segment elevation myocardial infarction, and unstable angina) during March 1 to April 30, 2018, January 1 to February 28, 2019, March 1 to April 30, 2019, and January 1 to February 29, 2020, as our control groups, as well as March 1 to April 30, 2020, as our study group of patients who presented during the COVID-19 pandemic. All procedures were performed at 2 hospitals in the Cleveland Clinic health system in Northeast Ohio: Cleveland Clinic Main Campus (Cleveland, Ohio) and Cleveland Clinic Akron General (Akron, Ohio). The incidence of stress cardiomyopathy was measured and compared across timelines.

### Diagnostic Criteria

Stress cardiomyopathy was diagnosed in accordance with the international Takotsubo syndrome diagnostic criteria in 2014 (ie, InterTAK diagnostic criteria).^4^ The criteria include transient left ventricular dysfunction presenting as apical ballooning or midventricular, basal, or focal wall-motion anomalies; an emotional, physical, or combined trigger may precede the disease onset (but is not obligatory); neurological disorders, as well as pheochromocytoma, may serve as triggers; new electrocardiographic anomalies are present (eg, ST-segment elevation, ST-segment depression, T wave inversion, and QTc prolongation); moderate elevation in cardiac biomarker levels (eg, troponin and creatine kinase) and significant elevation of brain natriuretic peptide; no evidence of myocarditis; and significant coronary artery disease may coexist. Findings on 12-lead electrocardiography, coronary arteriography, and echocardiography were interpreted and reported by Cleveland Clinic cardiologists. Clinical outcomes recorded in patients with stress cardiomyopathy included cardiac arrhythmias, hospital length of stay, 30-day rehospitalization, and overall mortality.

### Laboratory Confirmation

Testing for COVID-19 was performed using reverse transcription–polymerase chain reaction (RT-PCR), with samples collected from nasopharyngeal and throat swabs. COVID-19 testing in the study group was performed in patients meeting the previously published Cleveland Clinic COVID-19 testing criteria.^5^ All patients with stress cardiomyopathy in the study group were tested for COVID-19.

### Statistical Analysis

After assessing the distribution, continuous data were presented as medians and interquartile ranges (IQRs), and nominal data were presented as proportions. Continuous data across groups were compared using Kruskal-Wallis rank sum test, while categorical data were compared using Pearson χ^2^ test. The rate of stress cardiomyopathy among the pandemic and prepandemic periods was modeled using a Poisson regression model adjusting for overdispersion with a scale parameter. While each of the intervals studied was a 2-month period, the number of patients presenting with ACS and undergoing coronary arteriography differed among them. Thus, the number of patients served as an offset term (log transformed). Adjusted Wald method was used to compute 95% CIs of proportions. *P* values were 2-sided, and *P* < .05 was considered statistically significant. All statistical analyses were conducted in R statistical software version 3.6.2 (R Project for Statistical Computing). Data were analyzed from May to June 2020.

## Results

The final analysis included 1914 patients admitted to hospitals with ACS. A total of 1656 patients were admitted during the pre–COVID-19 period across 4 timelines, with 290 patients admitted from March to April 2018, 309 patients admitted from January to February 2019, 679 patients admitted from March to April 2019, and 278 patients admitted from January to February 2020. The COVID-19 period cohort included 258 patients admitted from March to April 2020. There were no significant differences between groups in the median (IQR) age (pre–COVID-19 period: 67 [59-74] years vs COVID-19 period: 67 [57-75] years; *P* = .56) or sex (pre–COVID-19 period: 1064 [66.1%] men vs COVID-19 period: 175 [67.8%] men; *P* = .43) (Table 1). Hypertension was the most frequently occurring comorbidity across all groups and was highest in the COVID-19 period (March-April 2018: 349 patients [89.5%]; January-February 2019: 259 patients [83.8%]; March-April 2019: 524 patients [77.2%]; January-February 2020: 229 patients [82.4%]; COVID-19 period: 232 patients [89.9%]; *P* < .001), followed by hyperlipidemia (March-April 2018: 294 patients [75.4%]; January-February 2019: 235 patients [76.1%]; March-April 2019: 461 patients [67.9%]; January-February 2020: 221 patients [79.5%]; COVID-19 period: 199 patients [77.1%]; *P* < .001). Compared with subgroups in other timelines, patients in the COVID-19 period had significantly lower median (IQR) initial troponin levels (March-April 2018: 0.28 [0.01-0.90] ng/mL; January-February 2019: 0.21 [0.01-1.01] ng/mL; March-April 2019: 0.40 [0.04-1.40] ng/mL; January-February 2020: 0.40 [0.07-1.38] ng/mL; COVID-19 period: 0.18 [0.03-0.50] ng/mL [to convert to micrograms per liter, multiply by 1]; *P* < .001) and peak troponin levels (March-April 2018: 2.42 [0.75- 6.27] ng/mL; January-February 2019: 1.48 [0.10-4.15] ng/mL; March-April 2019: 1.8 [0.21-6.95] ng/mL; January-February 2020: 3.10 [0.60-8.10] ng/mL; COVID-19 period: 0.7 [0.15-2.10] ng/mL; *P* < .001). Baseline characteristics of patients with stress cardiomyopathy stratified by pre–COVID-19 and COVID-19 periods are listed in Table 2. There were no significant differences in the baseline characteristics of patients with stress cardiomyopathy, except for hypertension (March-April 2018: 6 patients [100%]; January-February 2019: 5 patients [100%]; March-April 2019: 7 patients [58.3%]; January-February 2020: 5 patients [100%]; COVID-19 period: 19 patients [95.0%]; *P* = .01) and median (IQR) peak troponin levels (March-April 2018: 1.02 [0.92-3.12] ng/mL; January-February 2019: 1.30 [0.06-1.30] ng/mL; March-April 2019: 1.30 [0.15-2.64] ng/mL; January-February 2020: 1.80 [1.20-2.10] ng/mL; COVID-19 period: 0.30 [0.11-0.75] ng/mL; *P* = .03). All RT-PCR tests from patients during the COVID-19 pandemic were negative for COVID-19. There was a significant increase in the incidence of stress cardiomyopathy in patients presenting with ACS during the COVID-19 period, with a total of 20 patients (incidence proportion, 7.8%) compared with the pre–COVID-19 timelines, which ranged from 5 to 12 patients (incidence proportion range, 1.5%-1.8%) (Figure). Comparing the COVID-19 period with the combined prepandemic period, the rate ratio was 4.58 (95% CI, 4.11-5.11; *P* < .001). Adjusted rate ratios were similar in magnitude. When comparing the COVID-19 period to each prepandemic period individually, the rate ratios ranged from 4.31 (95% CI, 1.62-11.48) to 5.04 (95% CI, 2.02-12.55) (eTable in the Supplement).

**Table 1.** Baseline Characteristics of All Patients With Acute Coronary Syndrome Who Underwent Coronary Arteriography

Characteristic	Patients, No. (%)					*P* value
	Pre–COVID-19				COVID-19 (n = 258)^a^	
	March-April 2018 (n = 390)	January-February 2019 (n = 309)	March-April 2019 (n = 679)	January-February 2020 (n = 278)		
Age, median (IQR), y	67 (59-74)	67 (59-76)	67 (58-74)	66 (59-73)	67 (57-75)	.56
Men	252 (64.6)	211 (68.3)	458 (67.6)	173 (62.2)	175 (67.8)	.43
Comorbidity						
Hypertension	349 (89.5)	259 (83.8)	524 (77.2)	229 (82.4)	232 (89.9)	<.001
Diabetes	165 (42.3)	128 (41.4)	312 (46.1)	107 (38.5)	95 (36.8)	.06
Hyperlipidemia	294 (75.4)	235 (76.1)	461 (67.9)	221 (79.5)	199 (77.1)	<.001
Coronary artery disease	162 (41.5)	202 (65.4)	425 (62.6)	173 (62.2)	128 (49.6)	<.001
Atrial fibrillation	55 (14.1)	42 (13.6)	110 (16.2)	37 (13.3)	41 (15.9)	.70
Chronic kidney disease	55 (14.1)	38 (12.3)	92 (13.5)	26 (9.4)	40 (15.5)	.25
Asthma or COPD	72 (18.5)	51 (16.5)	67 (9.9)	35 (12.6)	19 (7.4)	<.001
COVID-19	NA	NA	NA	NA	0	
Troponin level, median (IQR), ng/mL						
Initial	0.28 (0.01-0.90)	0.21 (0.01-1.01)	0.40 (0.04-1.40)	0.40 (0.07-1.38)	0.18 (0.03-0.50)	<.001
High sensitivity initial	31.0 (14.0-132.5)	21.5 (12.5-131.2)	44 (14.3-222.8)	50.0 (26.0-302.0)	40.0 (18.5-152.0)	.30
Peak	2.42 (0.75- 6.27)	1.48 (0.10-4.15)	1.80 (0.21-6.95)	3.10 (0.60-8.10)	0.70 (0.15-2.10)	<.001
High sensitivity peak	35.0 (17.0-237.0)	21.5 (12.5-139.0)	50.0 (19.0-163.5)	228.0 (41.0-631.0)	54.0 (28.0-193.0)	.02
Pro-BNP, median (IQR), pg/mL	651.5 (209.2-3605.5)	675.0 (230.0-2608.0)	973.0 (223.3-4745.8)	441.0 (160.5-1990.5)	971.5 (316.8-3750.8)	.01
Ejection fraction, median (IQR)	55 (43-60)	55 (45-62)	55 (40-60)	55 (40.75-60)	54 (40-60)	.39
Ventriculogram, median (IQR)	55.0 (40.0-57.0)	40.0 (26.3-51.3)	44.0 (35.0-60.0)	37.5 (33.8-50.0)	45.0 (30.0-55.0)	.06
Stress cardiomyopathy, No. (%) [95% CI]	6 (1.5) [0.62-3.40]	5 (1.6) [0.58-3.84]	12 (1.8) [0.98-3.10]	5 (1.8) [0.65-4.26]	20 (7.8) [5.02-11.73]	<.001

Abbreviations: COPD, chronic obstructive pulmonary disease; COVID-19, coronavirus disease 2019; IQR, interquartile range; NA, not applicable; pro-BNP, pro–brain-type natriuretic peptide.

SI conversion factors: To convert troponin to micrograms per liter, multiply by 1; BNP to nanograms per liter, multiply by 1.

^a^ Defined as March 1 to April 30, 2020.

**Table 2.** Baseline Characteristics of Patients With Stress Cardiomyopathy

Characteristic	Patients, No. (%)					*P* value
	Pre COVID-19				COVID-19 [March-April 2020] (n = 20)^a^	
	March-April 2018 (n = 6)	January-February 2019 (n = 5)	March-April 2019 (n = 12)	January-February 2020 (n = 5)		
Age, median (IQR), y	65 (57-67)	60 (60-76)	69 (57-74)	56 (54-69)	63 (57-73)	.92
Men	3 (50.0)	0	3 (25.0)	1 (20.0)	7 (35.0)	.41
Comorbidities						
Hypertension	6 (100)	5 (100)	7 (58.3)	5 (100)	19 (95.0)	.01
Diabetes	1 (16.7)	1 (20.0)	4 (33.3)	2 (40.0)	3 (15.0)	.66
Hyperlipidemia	2 (33.3)	2 (40.0)	4 (33.3)	4 (80.0)	14 (70.0)	.14
Coronary artery disease	2 (33.3)	3 (60.0)	4 (33.3)	0	5 (25.0)	.32
Atrial fibrillation	0	2 (40.0)	1 (8.3)	1 (20.0)	3 (15.0)	.24
Chronic kidney disease	0	1 (20.0)	0	1 (20.0)	2 (10.0)	.48
Asthma or COPD	2 (33.3)	1 (20.0)	1 (8.3)	0	2 (10.0)	.45
COVID-19	NA	NA	NA	NA	0	
Troponin level, median (IQR), ng/mL						
Initial	0.11 (0.01-0.81)	0.40 (0.02-0.40)	0.16 (0.08-1.97)	0.50 (0.20-0.80)	0.05 (0.01-0.11)	.16
High sensitivity initial	NA	NA	NA	540.0 (540.0-540.0)	13.0 (11.5-21.5)	.18
Peak	1.02 (0.92-3.12)	1.3 (0.06-1.30)	1.30 (0.15-2.64)	1.80 (1.20-2.10)	0.30 (0.11-0.75)	.03
High sensitivity peak	NA	NA	NA	540.0 (540.0-540.0)	33.0 (21.0-84.0)	.18
Pro-BNP, median (IQR), pg/mL	4822.0 (4221.0-5369.0)	1313.0 (709.0-9730.0)	587.5 (223.2-5934.2)	3667.0 (3121.0-4988.0)	2322.0 (1509.0-3666.0)	.33
Ejection fraction, median (IQR)	32 (30-36)	30 (25-53)	28 (25-31)	30 (30-45)	30 (25-35)	.55
Ventriculogram, median (IQR)	NA	NA	37.5 (36.3-38.8)	15.0 (15.0-15.0)	15.0 (13.8-21.3)	.36
Mortality	0	0	0	1 (20.0)	1 (5.0)	.40
30-d rehospitalization	1 (16.7)	0	4 (36.4)	1 (20.0)	4 (22.2)	.59
Hospital length of stay, median (IQR), d	4 (3-4)	5 (3-6)	4 (4-8)	5 (4-5)	8 (6-9)	.006

Abbreviations: COPD, chronic obstructive pulmonary disease; COVID-19, coronavirus disease 2019; IQR, interquartile range; NA, not applicable; pro-BNP, pro–brain-type natriuretic peptide.

SI conversion factors: To convert troponin to micrograms per liter, multiply by 1; BNP to nanograms per liter, multiply by 1.

^a^ Defined as March 1 to April 30, 2020.

**Figure.**
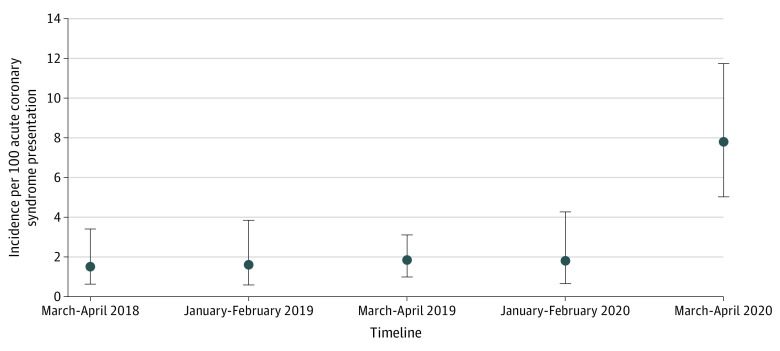
Incidence of Stress Cardiomyopathy per 100 Acute Coronary Syndrome Presentations During the Coronavirus Disease 2019 Pandemic and Prepandemic Periods March-April 2020 indicates the coronavirus pandemic period; dots, proportions calculated by adjusted Wald method; error bars, 95% CIs.

Patients with stress cardiomyopathy during the COVID-19 pandemic had a longer median (IQR) hospital length of stay compared with those hospitalized in the prepandemic period (COVID-19 period: 8 [6-9] days; March-April 2018: 4 [3-4] days; January-February 2019: 5 [3-6] days; March-April 2019: 4 [4-8] days; January-February: 5 [4-5] days; *P* = .006). There were no significant differences between the COVID-19 period and the overall pre–COVID-19 period in mortality (1 patient [5.0%] vs 1 patient [3.6%]; *P* = .81) or 30-day rehospitalization (4 patients [22.2%] vs 6 patients [21.4%]; *P* = .90).

## Discussion

To our knowledge, this cohort study is the first study to systematically investigate the association of the incidence of stress cardiomyopathy with the psychological, social, and economic stress associated with the COVID-19 pandemic. The principal finding of our analysis provides an insight into the increasing incidence of stress cardiomyopathy during the pandemic. The incidence of stress cardiomyopathy was significantly higher in patients presenting with ACS between March 1 and April 30, 2020, compared with 4 control groups across prepandemic timelines. The incidence of stress cardiomyopathy in the control groups was similar to that reported in the literature, ranging from 1.0% to 2.0% in patients presenting with acute myocardial infarction.^6,7,8^ The study group outcomes were similar to the control group with regard to mortality and 30-day rehospitalization. However, patients with stress cardiomyopathy hospitalized during the pandemic had a significantly longer hospital length of stay.

The association between stress cardiomyopathy and increasing levels of stress and anxiety has long been established.^4^ The psychological, social, and economic distress accompanying the pandemic, rather than direct viral involvement and sequelae of the infection, are more likely factors associated with the increase in stress cardiomyopathy cases. This was further supported by negative COVID-19 testing results in all patients diagnosed with stress cardiomyopathy in the study group.

There may still be an association of COVID-19 with Takotsubo-like cardiomyopathy. Few patients with Takotsubo syndrome with underlying COVID-19 have been reported in the literature.^2,3^ The mechanism behind this type of myocardial injury in patients with COVID-19 remains to be elucidated.

### Limitations

This study has some limitations. While our study examined patients from 2 hospitals within our health system, our sample represents the population of Northeast Ohio in the US. The results should be interpreted with caution when applied to other states or countries. Further research must examine the association of COVID-19 with the incidence of stress cardiomyopathy and study any temporal or regional differences. In addition, it is plausible that patients with ACS chose to avoid visiting a hospital facility amidst a pandemic, resulting in a sampling bias. Our study is also limited by the type of COVID-19 testing performed: RT-PCR is limited in its sensitivity (79%).^9^ This may have resulted in false-negative tests for our study group. However, none of the patients diagnosed with stress cardiomyopathy in the study group reported any symptoms suggestive of COVID-19–related illness.

## Conclusions

This cohort study found a significant increase in the incidence of stress cardiomyopathy during the COVID-19 pandemic. In addition, no patients with stress cardiomyopathy were found to have COVID-19, suggesting an indirect, psychological, social, and economic pandemic-related stress mechanism behind the disease process.

## Notes

Supplement. **eTable.** Incidence Rate Ratios of Stress Cardiomyopathy Comparing the COVID-19 Pandemic Period With Prepandemic Periods

## References

[1] Ćosić K, Popović S, Šarlija M, Kesedžić I Impact of human disasters and COVID-19 pandemic on mental health: potential of digital psychiatry. Psychiatr Danub. 2020;32(1):25-31. doi:10.24869/psyd.2020.25 32303026

[2] Sala S, Peretto G, Gramegna M, Acute myocarditis presenting as a reverse Tako-Tsubo syndrome in a patient with SARS-CoV-2 respiratory infection. Eur Heart J. 2020;41(19):1861-1862. doi:10.1093/eurheartj/ehaa286 32267502 PMC7184339

[3] Dabbagh MF, Aurora L, D’Souza P, Weinmann AJ, Bhargava P, Basir MB Cardiac tamponade secondary to COVID-19. JACC Case Rep. Published online April 23, 2020. doi:10.1016/j.jaccas.2020.04.009 32328588 PMC7177077

[4] Ghadri J-R, Wittstein IS, Prasad A, International expert consensus document on Takotsubo syndrome (Part I): clinical characteristics, diagnostic criteria, and pathophysiology. Eur Heart J. 2018;39(22):2032-2046. doi:10.1093/eurheartj/ehy076 29850871 PMC5991216

[5] Mehta N, Kalra A, Nowacki AS, Association of use of angiotensin-converting enzyme inhibitors and angiotensin II receptor blockers with testing positive for coronavirus disease 2019 (COVID-19). JAMA Cardiol. Published online May 5, 2020. doi:10.1001/jamacardio.2020.1855 32936273 PMC7201375

[6] Prasad A, Lerman A, Rihal CS Apical ballooning syndrome (Tako-Tsubo or stress cardiomyopathy): a mimic of acute myocardial infarction. Am Heart J. 2008;155(3):408-417. doi:10.1016/j.ahj.2007.11.008 18294473

[7] Akashi YJ, Goldstein DS, Barbaro G, Ueyama T Takotsubo cardiomyopathy: a new form of acute, reversible heart failure. Circulation. 2008;118(25):2754-2762. doi:10.1161/CIRCULATIONAHA.108.767012 19106400 PMC4893309

[8] Kurowski V, Kaiser A, von Hof K, Apical and midventricular transient left ventricular dysfunction syndrome (tako-tsubo cardiomyopathy): frequency, mechanisms, and prognosis. Chest. 2007;132(3):809-816. doi:10.1378/chest.07-0608 17573507

[9] Li Y, Xia L Coronavirus disease 2019 (COVID-19): role of chest CT in diagnosis and management. AJR Am J Roentgenol. 2020;214(6):1280-1286. doi:10.2214/AJR.20.22954 32130038

