# Supplementary material for: Incidence of Stress Cardiomyopathy During the Coronavirus Disease 2019 Pandemic
Source: JAMA Netw Open. 2020 Jul 9;3(7):e2014780. doi: 10.1001/jamanetworkopen.2020.14780 (PMC7348683; doi:10.1001/jamanetworkopen.2020.14780)
Supplement: Supplement. — eTable. Incidence Rate Ratios of Stress Cardiomyopathy Comparing the COVID-19 Pandemic Period With Prepandemic Periods [file jamanetwopen-3-e2014780-s001.pdf]

Supplementary Online Content

Jabri A, Kalra A, Kumar A, et al. Incidence of stress cardiomyopathy during the coronavirus disease 2019 pandemic. *JAMA Netw Open*. 2020;3(7):e2014780. doi:10.1001/jamanetworkopen.2020.14780

eTable. Incidence Rate Ratios of Stress Cardiomyopathy Comparing the COVID-19 Pandemic Period With Prepandemic Periods

This supplementary material has been provided by the authors to give readers additional information about their work.

© 2020 Jabri A et al. *JAMA Network Open.*

eTable. Incidence Rate Ratios of Stress Cardiomyopathy Comparing the COVID-19 Pandemic Period With Prepandemic Periods

	**Risk Ratio**	**95% CI**	***P* value**
**Unadjusted Models**			
COVID19 vs. all pre-COVID19	4.58	(4.11, 5.11)	<.0001
COVID19 (Mar - Apr 2020) vs. Jan - Feb 2020	4.31	(1.62, 11.48)	0.004
COVID19 (Mar - Apr 2020) vs. Mar - Apr 2019	4.39	(2.14, 8.97)	<.0001
COVID19 (Mar - Apr 2020) vs. Jan - Feb 2019	4.79	(1.80, 12.77)	0.002
COVID19 (Mar - Apr 2020) vs. Mar - Apr 2018	5.04	(2.02, 12.55)	0.0005
**Adjusted Models**			
HTN: COVID19 vs. all pre-COVID19	5.01	(4.55, 5.51)	<.0001
HLD: COVID19 vs. all pre-COVID19	4.68	(4.05, 5.40)	<.0001
CAD: COVID19 vs. all pre-COVID19	4.79	(4.31, 5.32)	<.0001
Asthma/COPD: COVID19 vs. all pre-COVID19	4.16	(3.87, 4.47)	<.0001
CAD = coronary artery disease, CI = confidence interval, COPD = chronic obstructive pulmonary disease, HLD = hyperlipidemia, HTN = hypertension			

© 2020 Jabri A et al. *JAMA Network Open.*
